# Ocean Acidification Effects on Atlantic Cod Larval Survival and Recruitment to the Fished Population

**DOI:** 10.1371/journal.pone.0155448

**Published:** 2016-08-23

**Authors:** Martina H. Stiasny, Felix H. Mittermayer, Michael Sswat, Rüdiger Voss, Fredrik Jutfelt, Melissa Chierici, Velmurugu Puvanendran, Atle Mortensen, Thorsten B. H. Reusch, Catriona Clemmesen

**Affiliations:** 1 Evolutionary Ecology of Marine Fishes, GEOMAR Helmholtz Centre for Ocean Research, Kiel, Germany; 2 Sustainable Fisheries, Department of Economics, University of Kiel, Kiel, Germany; 3 Biological Oceanography, GEOMAR Helmholtz Centre for Ocean Research, Kiel, Germany; 4 Department of Biology, Norwegian University of Science and Technology, Trondheim, Norway; 5 Institute of Marine Research, Tromsø, Norway; 6 Nofima AS, Tromsø, Norway; Stony Brook University, UNITED STATES

## Abstract

How fisheries will be impacted by climate change is far from understood. While some fish populations may be able to escape global warming via range shifts, they cannot escape ocean acidification (OA), an inevitable consequence of the dissolution of anthropogenic carbon dioxide (CO_2_) emissions in marine waters. How ocean acidification affects population dynamics of commercially important fish species is critical for adapting management practices of exploited fish populations. Ocean acidification has been shown to impair fish larvae’s sensory abilities, affect the morphology of otoliths, cause tissue damage and cause behavioural changes. Here, we obtain first experimental mortality estimates for Atlantic cod larvae under OA and incorporate these effects into recruitment models. End-of-century levels of ocean acidification (~1100 μatm according to the IPCC RCP 8.5) resulted in a doubling of daily mortality rates compared to present-day CO_2_ concentrations during the first 25 days post hatching (dph), a critical phase for population recruitment. These results were consistent under different feeding regimes, stocking densities and in two cod populations (Western Baltic and Barents Sea stock). When mortality data were included into Ricker-type stock-recruitment models, recruitment was reduced to an average of 8 and 24% of current recruitment for the two populations, respectively. Our results highlight the importance of including vulnerable early life stages when addressing effects of climate change on fish stocks.

## Introduction

The understanding of the effect of global change on fish populations is critical for sustainable exploitation and management of fisheries [1]. Ocean warming has already triggered poleward range shifts of many marine fish populations caused by their thermal tolerance [2–4]. However, higher latitudes provide no refuge with respect to the concomitant pH decline, caused by the dissolution of the major greenhouse gas CO_2_ in ocean waters. This “other CO_2_ problem”, also dubbed ocean acidification (OA) [5], is an inevitable consequence of anthropogenic release of CO_2_. The potential consequences of ocean acidification on commercially important fish populations are intensely debated [6,7], but currently unresolved since data on population-level processes, e.g. recruitment to the stock, are almost entirely lacking [8–10].

Adult fishes have been shown to tolerate extreme CO_2_ concentrations of up to 16,000 μatm [11], which led to the premature conclusion that fishes are less vulnerable to ocean acidification than for example calcifying organisms [12]. However, it is becoming increasingly evident that early life stages such as eggs and larvae are more susceptible to decreased ocean pH [7,13]. This is partly due to insufficient acid-base regulation prior to the formation of gills [14]. Recent studies have shown a diverse range of impacts of predicted future CO_2_ concentrations on larval fish, particularly on sensory abilities like olfaction [15], behaviour [16,17], otoliths [18–20], development, tissue and organ structure [13,21]. Studies also found effects on survival of eggs, more specifically hatching success [22], and survival of very early larval stages [7,23]. Other studies were not able to find an effect on survival [24,25].

Survival, however, is the most important parameter to assess recruitment, thus of paramount importance for stock management. Recruitment to an exploited fish stock is defined as that point of time when a year-class enters the fished population, i.e. at an age of 1 year in the case of Western Baltic cod, and at an age of 3 years in Barents Sea cod. Here we assess larval mortality as a key variable to predict population growth and size [26,27] in Atlantic cod (*Gadus morhua*, L.) under end-of-century CO_2_ concentrations. This is one of the most important species for commercial fisheries of the North Atlantic, It is of particular importance since landings of many cod stocks have decreased in the past decades with some stocks collapsing [28]. Any additional source of mortality, particularly one with a trend, should therefore be closely monitored and incorporated into management strategies.

We designed two experiments, in which the survival of cod larvae was quantified in direct response to increased *p*CO_2_ levels as predicted for the end of the century. Atmospheric CO_2_ concentrations have been continuously rising since the beginning of industrialisation and are currently exceeding 400 μatm. A third of the excess CO_2_ is absorbed by the world’s oceans, resulting in ocean acidification, leading to an estimated decrease in pH of 0.4 units (*p*CO_2_ ~ 1,000 μatm) by the end of the century [5,29,30]. Eggs and larvae from the Western Baltic cod stock, caught in the Øresund, and from the Arcto-Norwegian Barents Sea cod stock were kept under control (~400–500 μatm) and high CO_2_ (~1100 μatm) concentrations in two separate experiments until 25 and 22 days post-hatching (dph) respectively and survival was monitored closely.

## Methods and Materials

For the Western Baltic experiment, adult cod were caught in the Øresund (55°58’N, 12°38’E) in March 2013 and strip-spawned. An equal volume of eggs was placed in 90 L rearing tanks at the Sven Lovén Centre, Kristineberg, Sweden. Three tanks were kept under ambient CO_2_ concentrations of 426 ± 47 μatm and three tanks were kept under increased CO_2_ conditions of 1033 ± 255 μatm. The temperature was kept constant at 7°C and the light regime was matched weekly to the ambient sun rise and sun set. After hatching the larvae were fed with natural plankton from the Gullmars Fjord under green water conditions with *Nannochloropsis*. (Food density estimates are given in Table A in S1 File). Survival was measured daily by collecting and counting all dead larvae from the bottom of the tanks. Initial number of larvae (on average ~800 larvae per tank) was then back-calculated to calculate survival in percentage. It was shown in separate experiments that dead larvae were easily found even after more than 24 hours post mortem in the tanks.

For the Barents Sea cod experiment adult fish were caught alive in the Barents Sea (70°15’N, 19°00’E) in March 2014 and transferred to the National Cod Breeding Centre, Tromsø. They were kept in large breeding tanks (25 m3) with flow-through from the fjord and at weekly matched ambient light regimes. All naturally produced eggs were collected using collectors behind the surface skimmer outflow. These were transferred to incubators with either ambient (503 ± 89 μatm CO2) or increased CO2 (1179 ± 87 μatm) concentrations. After peak hatch (more than 50% eggs hatched), 11,000 larvae were transferred into each of twelve 190 L rearing tanks with a constant flow-through of water from a common header tank. For the egg incubation and the start of the experiment the temperature was set to 6°C and was later raised to 10°C in all tanks at constant light conditions (24h). Larvae were fed with *Nannochloropsis* and *Brachionus* at different intervals for the high and the low food treatment (seven compared to three times daily), while the prey concentrations per feeding remained the same for both treatments. (For information on the feeding conditions, see Table B in S1 File). It should be noted, that even though the low food treatment only provided a fraction of the total amount of prey of the high food treatment, it is likely still higher than prey densities, which the larvae would experience in the field. However, this is difficult to compare, since we provided very high densities for short periods at the feeding times, which were then washed out of the tanks again. Therefore no steady density of prey was provided, but during feeding times prey densities were extremely high. This allowed for the exclusion of density and competition effects, which may have otherwise arisen due to different larval densities in the different treatments. Larvae in one tank in the ambient CO2 treatment were abruptly lost over night, due to an unknown factor, resulting in six replicates for the high CO2 treatment and five for the ambient treatment, each divided equally into the high and low food treatment. Starting on 8 dph survival was measured every four to six days by calculating the density of the larvae in the tanks. Five times 0.8 l of water was sampled from each tank over the whole water column using a pipe that could be closed at the bottom and the larvae contained in the pipe were subsequently counted in each sub sample. Prior to sampling an even distribution of larvae in the rearing tanks was achieved by increasing the aeration.

For both experiments the mean mortality coefficient was calculated after non-linear curve fitting of a negative exponential function for each replicate tank. Mean daily mortality rates (in percentage per day) were compared between treatments using a t-test (Western Baltic stock) and a two-way ANOVA (Barents Sea stock) after appropriate data transformation to achieve homogeneity of variances.

Ambient and increased CO_2_ levels were achieved by controlling the pH values in a header tank with pH sensors connected to an IKS computer system. If the values deviated from the set target pH a magnetic valve opened automatically, which allowed a pulse of CO_2_ from a CO_2_ bottle to be injected into the header tank. The volume of the header tank ensured a thorough mixing and equilibration of CO_2_ before the water entered the rearing tank thereby assuring constant conditions in the rearing tanks. The pH was furthermore manually checked every day in the rearing tanks with a separate pH sensor (WTW pH/Cond 340i/3320). Water chemistry, including DIC and alkalinity, was tested at the beginning and the end of the experiment for the Western Baltic cod experiment and weekly for the Barents Sea cod experiment based on the Best Practices Guide [31]. Further details regarding methods and carbon chemistry analysis are available in the Supporting Information.

All experiments were carried out in accordance to the national rules and regulations at the site of the experiments and all efforts where undertaken to minimize stress and suffering of the animals. Issues for work on vertebrate animals were obtained for each experiment and location. For the experiment in Kristineberg with the Western Baltic cod the ethics permit number is 332–2012 issued by the Swedish Board of Agriculture (Jordbruksverket). For the experiment in Tromsø on the Barents Sea cod the ethics permit number is FOTS ID 6382, issued by the Norwegian Animal Research Authority (Forsøksdyrutvalget). In accordance with these permits animals were euthanized after the experiment or whenever some were taken out for density measurements using Tricaine methanesulfonate (MS222). No endangered or protected species were used in these experiments and no other special permits were necessary.

### Population level effects

Considering the potential impact of ocean acidification on fisheries requires scaling from physiological responses to population-level processes. A simple way is to consider how ocean acidification could modify the parameters of growth, mortality and reproduction in a single-species. Here we concentrate on the modification of the parameters of the stock-recruitment relationship in an age-structured fishery model.

The effect of ocean acidification was assessed by modifying the density-independent parameter *α* of a Ricker type stock recruitment relationship. Ocean acidification causes a higher larval mortality rate. This leads to a density-independent mortality rate a caused by acidification. In the baseline scenario (no acidification) *a* = 0, while in the acidification scenarios, e^-a^ is the fraction of larvae surviving the effect of acidification. We used our experimental data to quantify this effect, and to compare scenarios (See Supporting Information). We used ICES data for Western Baltic cod for the years 1970 to 2014 and for Arcto-Norwegian cod for the years 1946–2014 to estimate the stock-recruitment relationship for the baseline scenario. We assume log-normal auto-correlated errors, and estimated the model. (Further details regarding the recruitment models are available as Supporting Information.) Because the severity of ocean acidification induced mortality on recruitment depends on the duration of the additional mortality, two developmental stages were chosen as termination for the enhanced mortality [20]. Based on the experimental temperatures at day 23 days post hatching the larval gut has reached its typical spiral form (and potentially altered function) while at 30 dph gills become visible on the gill arches. These two time points were used to evaluate the effect of increased mortality on recruitment success assuming the same mortality estimates until 30 dph as shown in the experiments until 22 dph and 25 dph. Mortality during the recruitment process consists of both density-independent and density-dependent effects. For simplicity we assume that the effect of ocean acidification on the survival will only influence the density-independent mortality during the recruitment phase potentially biasing the data to be on the conservative side.

## Results

**Fig 1:**
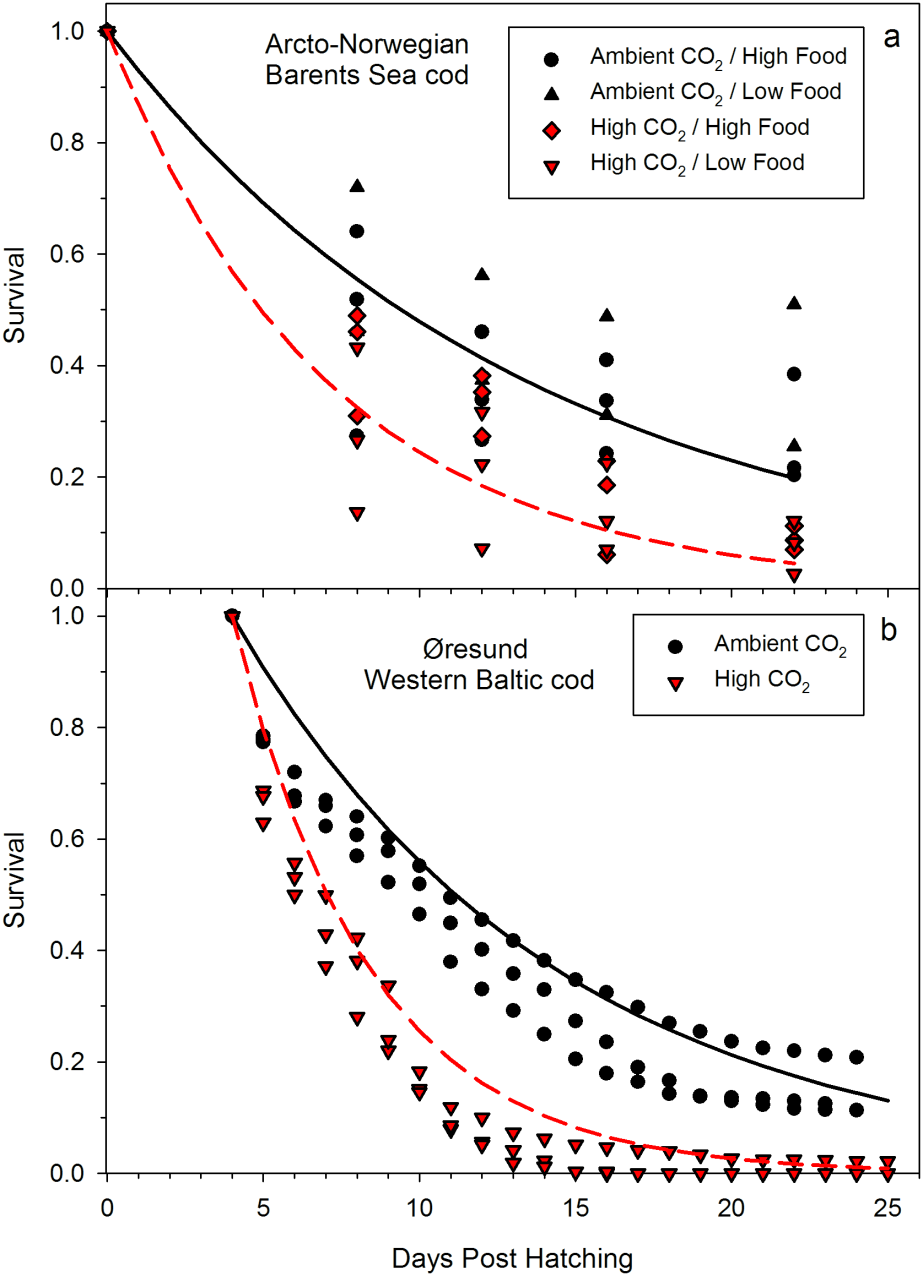
Effect of increased CO_2_ on early life survival of *Gadus morhua* from (a) Barents Sea cod (b) Western Baltic cod. Each symbol represents the value of one replicate tank. Lines depict the number of survivors according to the fitted negative exponential function.

The effect of CO_2_ was consistent among stocks and experimental conditions, i.e. different feeding conditions. At increased CO_2_ concentrations the daily mortality rates had approximately doubled in both experiments, from 7 to 13% in the Barents Sea stock (Fig 1a) and from 9.2 to 20.4% in the Western Baltic Sea stock (Fig 1b) (Western Baltic experiment, T-test, t = -3.749, df = 2.41, *p* = 0.024; Barents Sea experiment Two-way ANOVA F = 8.434, df = 1, *p* = 0.023). In the Barents Sea experiment the food density had no detectable effect on mortality rate, neither as main effect nor in interaction with the CO_2_-treatment (for additional statistics, see Tables C and D in S1 File). Cod larvae therefore appear to be negatively affected by ocean acidification even when *ad libitum* prey densities should ensure that energy is available for potential acid-base regulation mechanisms.

**Fig 2:**
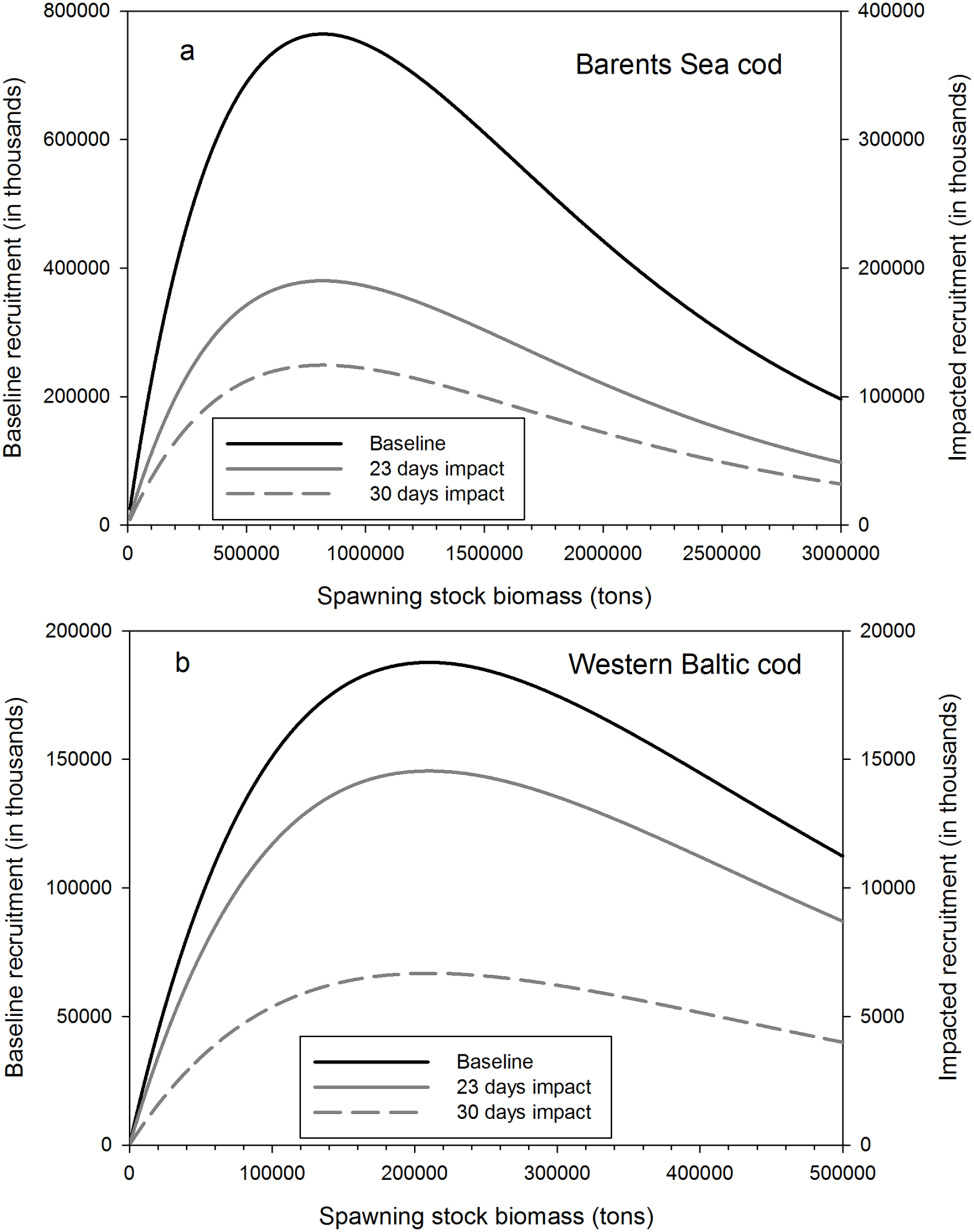
Recruitment functions under baseline and under ocean acidification scenarios for (a) the Barents Sea cod and (b) the Baltic Sea cod. The baseline scenario is based on no OA and spawning stock biomass at ICES precautionary biomass levels (B_PA_) in dependence of the duration of OA-induced mortality. For better visualization a different scaling on the second y-axes was chosen for the impacted recruitment.

**Fig 3:**
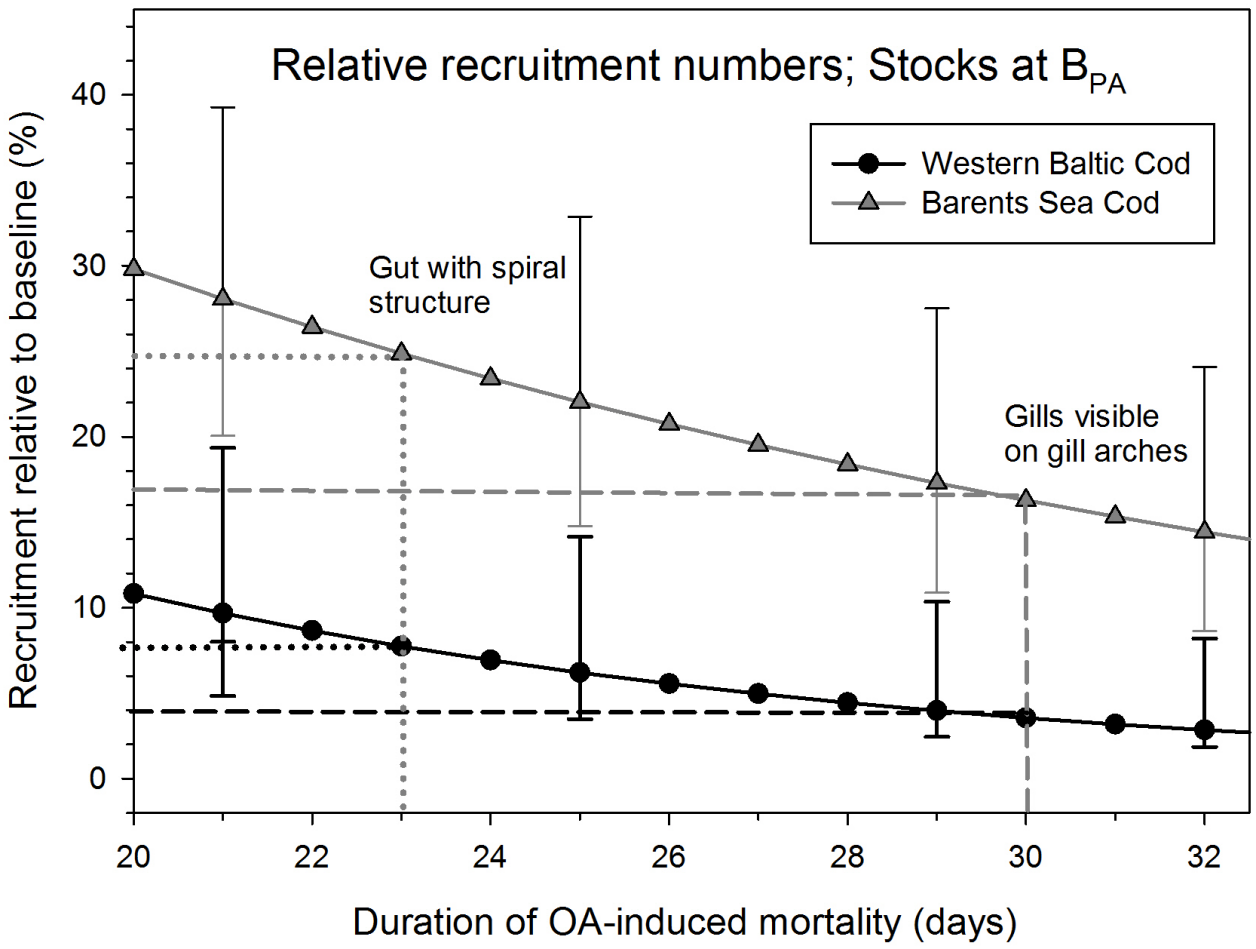
Population recruitment under ocean acidification (OA) for Western Baltic cod (black line and symbols) and Barents Sea cod (grey line and symbols). Recruitment is given relative to a baseline scenario of no OA and spawning stock biomass at ICES precautionary biomass levels (B_PA_) in dependence of the duration of OA-induced mortality. Two important points in larval development are highlighted. Standard deviations displayed only for selected days to improve readability.

Next, the experimentally assessed larval mortality rates were incorporated into a Ricker-type stock-recruitment model that was parametrized for the two studied cod populations. We concentrated on altering the larval mortality in order to evaluate the overall stock-recruitment relationship to assess their effects on population dynamics (for details see Supporting Information). The model results show that for both mortality scenarios increased larval mortality due to ocean acidification will reduce recruitment substantially. Recruitment levels will be reduced on average to only 8% of the baseline scenario in the case of Western Baltic cod for ocean acidification-induced mortality periods of 23 days (and 4% for a mortality period of 30 days), and to 24.5% (and 17% respectively) in Arcto-Norwegian cod (Figs 2 and 3).

## Discussion

Under realistic scenarios of end-of-century ocean acidification, early larval survival of cod was significantly reduced in two separate experiments with two different Atlantic cod stocks. Results were consistent under different feeding regimes and strongly suggest that there is a severe effect of ocean acidification on Atlantic cod larvae and recruitment.

Mass spawning fishes such as cod have many offspring with low survival probability in nature. The salient question is whether our experimental conditions provide appropriate controls with reasonable natural mortality levels. Larval survival rates are naturally low even under ambient CO_2_ concentrations and optimal feeding conditions. The mortality is mainly caused by the difficulty in a successful first feeding once the yolk sac is absorbed [27]. Other studies find similar mortality rates as our control values in the two experiments during early larval development [32,33]. Survival of larvae in our experiment from the Western Baltic stock was lower than for the Barents Sea stock, since they were fed with natural plankton in concentrations as provided by the fjord, while the larvae from the Barents Sea stock were kept under aquaculture conditions aiming for the production of the highest numbers of fingerlings for stocking of industrial scale production net pens.

Larval fish survival under ocean acidification has so far been shown in only one other study by Baumann *et al*. (2012) [7], albeit in a non-commercial fish species, the Atlantic silverside (*Menidia menidia*). In their study reduced larval survival was observed at 1100 *ppm*, a level of ocean acidification, which is predicted to occur globally at the start of the next century under the IPCC RCP 8.5, during the first week post hatch. Chambers *et al*. (2013) [22] found a decreased hatching success (reflecting embryonic development) of the summer flounder by 50% under 1860 *ppm*. This is a realistic ocean acidification level for the environment of this species within this century, even though values on a global average are predicted to be lower. Munday *et al*. (2015) [25] found no effect on the survival of yellowtail kingfish larvae. Other studies, like Munday *et al*. (2009b) [24]; Franke & Clemmesen (2011) [34]; Frommel *et al*. (2013) [35]; Hurst *et al*. (2013, 2015) [36,37], have addressed hatching success and have not seen any effects of ocean acidification. We are confident that this does not necessarily indicate that these species will not be affected or that our results present a contradiction. It is well known that early life stages of marine fish go through several bottlenecks with high mortalities during development and that different populations of the same species can react differently to CO_2_ stress [35]. Our results show that the first days and weeks after hatching are a vulnerable phase to ocean acidification. So far studies on tropical fish have not seen an ocean acidification effect on survival [38]. This is not surprising, since early development in the studied species is very different from temperate fish and newly hatched larvae are further developed and physiologically more competent thus less vulnerable to physiological stressors. Furthermore the study by Munday *et al*. (2011), and other studies like Hurst *et al*. (2013), only quantified survival at a single day, which may not have been the final day of any additional mortality. Additionally, even if this was an end-point measurement, it does not allow for calculations of mortality rates.

One factor that this study is not taking into account is possibility that parental exposure to the high CO_2_ environment could limit the adverse effects of ocean acidification. This kind of transgenerational adaptation has been shown to mediate negative growth effects of OA in tropical reef fish [39]. However since most commercially important fish species are quite large and temperate fish species reach sexual maturity late, it will be difficult to perform experiments with long parental exposure time. Furthermore it cannot be ruled out, that ocean acidification might also have an additional negative effect on gonadal development in adult fishes, which might further reduce recruitment potential.

Range shifts are responses of many fish populations to track the poleward movement of their thermal range [2]. Unfortunately, this may exacerbate direct CO_2_ effects identified here, since oceanic waters in higher latitudes will take up more CO_2_ due to higher solubility and experience lower carbonate saturation [40]. Previously, ocean acidification has been shown to affect marine fish larvae’s sensory abilities, morphology of the otoliths, cause tissue damage and behavioural differences [13,17,18,19,21].

Here we give the first demographic estimates for Atlantic cod under realistic end-of-century ocean acidification levels which are urgently needed to estimate whether these exploited fish populations could potentially expect population declines as a direct consequence of ocean acidification. The estimated recruitment declines shown are severe, of similar magnitude as population collapses due to overfishing [41] and have highly significant implications for the governance of exploited fish populations. We show that indeed, increased mortality will affect recruitment at the population level, demonstrating that any future management of exploitation must directly consider effects induced by global change.

## Supporting Information

S1 File Supporting Information on experimental set-up, carbon chemistry, statistics and recruitment modelling. (DOCX)
