# Supplementary material for: Ocean Acidification Effects on Atlantic Cod Larval Survival and Recruitment to the Fished Population
Source: PLoS One. 2016 Aug 23;11(8):e0155448. doi: 10.1371/journal.pone.0155448 (PMC4995109; doi:10.1371/journal.pone.0155448)
Supplement: S1 File — (DOCX) [file pone.0155448.s001.docx]

# Supporting Information

**Experimental set-up**

For the Western Baltic experiment, adult cod were caught in the Øresund (55°58’N, 12°38’E) in March 2013 and strip-spawned to create fifteen families (3 females x 5 males). An equal volume of eggs was placed in 90 L rearing tanks at the Sven Lovén Centre, Kristineberg, Sweden. Three tanks were kept under ambient CO_2_ concentrations of 426 ± 47 µatm and three tanks were kept under increased CO_2_ conditions of 1033 ± 255 µatm. The temperature was kept constant at 7°C and the light regime was matched weekly to the ambient sun rise and sun set. After hatching the larvae were fed with natural plankton from the Gullmars Fjord (daily concentrations are shown in Table A) and with *Nannochloropsis*.

SI Table A. Feeding densities for the Western Baltic stock.

|  | Kristineberg Natural Plankton | | | |
| --- | --- | --- | --- | --- |
| dph | *Nannochloropsis* added | First Daily Feeding (prey org ml^-1^ feeding^-1^) | Second Daily Feeding (prey org ml^-1^ feeding^-1^) | Third Daily Feeding (prey org ml^-1^ feeding^-1^) |
| 1 | yes | 0 | 0 | 0 |
| 2 | yes | 0 | 0 | 0 |
| 3 | yes | 0 | 0 | 0 |
| 4 | yes | 0 | 0 | 0 |
| 5 | yes | 0.18 | 0 | 0 |
| 6 | yes | 0 | 0 | 0 |
| 7 | yes | 0.34 | 0 | 0 |
| 8 | yes | 0.51 | 0 | 0 |
| 9 | yes | 0.20 | 0.18 | 0 |
| 10 | yes | 0.18 | 0.34 | 0 |
| 11 | yes | 0.70 | 0 | 0 |
| 12 | yes | 0.70 | 0.67 | 0 |
| 13 | yes | 0.37 | 0.34 | 0 |
| 14 | yes | 0.43 | 0.48 | 0 |
| 15 | yes | 0.65 | 0.55 | 0.44 |
| 16 | yes | 0.18 | 0.40 | 0 |
| 17 | yes | 0.12 | 0.17 | 0.20 |
| 18 | yes | 0.24 | 0.34 | 0 |
| 19 | yes | 0.13 | 0.19 | 0 |
| 20 | yes | 0.28 | 0.15 | 0.18 |
| 21 | yes | 0.11 | 0.08 | 0 |
| 22 | yes | 0.17 | 0.12 | 0.15 |
| 23 | yes | 0.21 | 0.08 | 0 |
| 24 | yes | 0.12 | 0 | 0 |
| 25 | yes | 0.27 | 0.86 | 0 |

Survival was measured daily by collecting all dead larvae from the bottom of the tanks and counting these. Initial number of larvae was then back-calculated to calculate survival in percentage. It was shown in separate experiments that dead larvae were easily found even after more than 24 hours in the tanks.

For the Barents Sea cod experiment adult fish were caught alive in the Barents Sea (70°15’N, 19°00’E) in March 2014 and transferred to the National Cod Breeding Centre, Tromsø. They were kept in large breeding tanks (25 m^3^) and all produced eggs were collected from the outflow. These were transferred to incubators with either ambient
(503 ± 89 µatm) or increased CO_2_ (1179 ± 87 µatm) concentrations. After peak hatch (more than 50% eggs hatched), 11,000 larvae were transferred into each of twelve 190 L rearing tanks with a constant flow-through of water from a common header tank. For the egg incubation and the start of the experiment the temperature was set to 6°C and was later raised to 10°C in all tanks at constant light conditions (24h). Larvae were fed with enriched rotifers*.* Densities and number of daily feedings can be found in Table B.

SI Table B. Feeding densities for the Barents Sea stock.

| dph | *Nannochloropsis* added | prey organisms ml^-1^ feeding^-1^ | Low Food  number of daily feedings | Low Food Prey organisms per day per tank (ml) | High Food  number of daily feedings | High Food  Prey organisms per day per tank (ml) |
| --- | --- | --- | --- | --- | --- | --- |
| 1 | yes | 3.2 | 7 | 4.27 | 7 | 4.27 |
| 2 | yes | 3.2 | 7 | 4.27 | 7 | 4.27 |
| 3 | yes | 3.2 | 7 | 4.27 | 7 | 4.27 |
| 4 | yes | 3.2 | 7 | 4.27 | 7 | 4.27 |
| 5 | yes | 5.5 | 7 | 7.35 | 7 | 7.35 |
| 6 | yes | 5.5 | 7 | 7.35 | 7 | 7.35 |
| 7 | yes | 5.5 | 7 | 7.35 | 7 | 7.35 |
| 8 | yes | 5.5 | 7 | 7.35 | 7 | 7.35 |
| 9 | yes | 5.5 | 7 | 7.35 | 7 | 7.35 |
| 10 | yes | 5.5 | 7 | 7.35 | 7 | 7.35 |
| 11 | yes | 5.5 | 7 | 7.35 | 7 | 7.35 |
| 12 | yes | 5.5 | 7 | 7.35 | 7 | 7.35 |
| 13 |  | 5.5 | 7 | 7.35 | 7 | 7.35 |
| 14 |  | 5.5 | 3 | 3.15 | 7 | 7.35 |
| 15 |  | 5.5 | 3 | 3.15 | 7 | 7.35 |
| 16 |  | 5.5 | 3 | 3.15 | 7 | 7.35 |
| 17 |  | 5.5 | 3 | 3.15 | 7 | 7.35 |
| 18 |  | 5.5 | 3 | 3.15 | 7 | 7.35 |
| 19 |  | 5.5 | 3 | 3.15 | 7 | 7.35 |
| 20 |  | 5.5 | 3 | 3.15 | 7 | 7.35 |
| 21 |  | 5.5 | 3 | 3.15 | 7 | 7.35 |
| 22 |  | 5.5 | 3 | 3.15 | 7 | 7.35 |

Larvae in one tank in the ambient CO_2_ treatment were abruptly lost over night, due to an unknown factor, resulting in six replicates for the high CO_2_ treatment and five for the ambient treatment. Starting on 8 dph the survival was measured every four to six days by calculating the density of the larvae in the tanks, sampling five times 0.8 L of water from the tanks over the whole water column using a pipe that could be closed at the bottom and then counting larvae in this sub sample. An even distribution was achieved by increasing the air inflow through the aeration stones.

**Set-up and determination of the CO_2_-system**

Ambient and high CO_2_ levels were achieved by controlling the pH values in a header tank with pH probes connected to a computer monitoring system (IKS-aquastar). If the values deviated from the set target pH a magnetic valve was opened, which allowed a pulse of CO_2_ from a CO_2_ bottle to go into the header tank. The volume of the header tank ensured a thorough mixing and equilibration of CO_2_ before the water entered the rearing tank thereby assuring constant conditions in the rearing tanks. The pH was furthermore manually checked every day in the rearing tanks with a separate pH probe (WTW pH/Cond 340i/3320). Water chemistry, including C_T_ (total carbon) and A_T_ (total alkalinity), was tested at the beginning and the end of the experiment for the Western Baltic cod experiment and weekly for the Barents Sea cod experiment based on the Best Practices Guide(*1*).

Analytical methods for C_T_ (total carbon) and A_T_ (total alkalinity) determination in seawater samples are fully described in *Dickson et al.,* (2007)(*2*). Briefly, C_T_ was determined using gas extraction of acidified sample followed by coulometric titration and photometric detection using a Versatile Instrument for the Determination of Titration carbonate (VINDTA 3C, Marianda, Germany). A_T_ was determined in water column samples from potentiometric titration with 0.1 N hydrochloric acid using a Versatile Instrument for the Determination of Titration Alkalinity (VINDTA 3C, Marianda). The average standard deviation for C_T_ and A_T_, determined from replicate sample analyses from one sample, was within ±1 μmol kg^-1^. The accuracy of the measurements were ensured by routine analyses of Certified Reference Materials (CRM, provided by A. G. Dickson, Scripps Institution of Oceanography, USA) and was better than ±1 μmol kg^−1^ and ±2 μmol kg^−1^ for C_T_ and A_T_, respectively.

We used C_T_, A_T_, salinity, and temperature, for each sample as input parameters in a CO_2_-chemical speciation model (CO2SYS program(*3*)) to calculate all the other parameters in the CO_2_-system such as pH in situ, CO_2_ fugacity and partial pressure (*f*CO_2_, *p*CO_2_), carbon dioxide concentration ([CO_2_]) and carbonate-ion concentration ([CO_3_^2-^]), and calcium-carbonate saturation states in the water column (Ω) for aragonite (Ω_Ar_) and calcite (Ω_Ca_), We used the total hydrogen-ion scale (pH_T_), the HSO_4_^-^ dissociation constant of Dickson, 1990(*4*) and the CO_2_-system dissociation constants (K*_1_ and K*_2_) estimated by Mehrbach et al., 1973(*5*) refit by Dickson and Millero (1987)(*6*). Mean values and standard deviation of *p*CO_2_ in the Western Baltic cod experiment were 1033 +- 255 µatm for the high and 426 +- 47 µatm for the ambient treatment which is equivalent to a pH value (total scale at in situ temperature) of 7.76 +- 0.09 for the high and 8.17 +- 0.03 at ambient conditions. Mean values and standard deviation of *p*CO_2_ in the Barents Sea cod experiment were 1179 +- 87 µatm for the high and 503 +- 89 µatm for the ambient treatment which is equivalent to a pH value (total scale at in situ temperature) of 7.61 +- 0.03 for the high and 7.90 +- 0.15 at ambient conditions.

**Statistics**

Data were cubic-root transformed to achieve variance homogeneity, assessed with Bartlett’s test. Results are shown in Table C and D.

SI Table C. Statistics for the Western Baltic cod stock.

| **Source of variation** | **Degrees of freedom** | **t-ratio** | **p-value** |
| --- | --- | --- | --- |
| CO_2_ | 2.41 | -3.749 | 0.024 |

SI Table D. Statistics for the Barents Sea cod stock.

| **Source of variation** | **Degrees of freedom** | **F** | **p-value** |
| --- | --- | --- | --- |
| CO_2_ | 1 | 8.434 | 0.023 |
| Food | 1 | 0.06 | 0.814 |
| CO_2_*Food | 1 | 2.325 | 0.171 |

**Recruitment model**

Experimental studies, like the one presented here, mostly refer to effects of ocean acidification on physiological processes. Considering the potential impact of ocean acidification on fisheries requires scaling from physiological responses to population- and ecosystem-level processes. A simple way is to consider how ocean acidification could modify the parameters of growth, mortality and reproduction in a single-species model(*7*). Here we concentrate on the modification of the parameters of the stock-recruitment relationship in an age-structured fishery model. For visualization purposes we choose recruitment at the management target of precautionary biomass levels (B_PA_) as given by ICES^8,9^.

We assume that egg production in $N_{0}$, is proportional to spawning stock biomass, SSB, i.e.$N_{0}=f \text{SSB}$, where *f* is the net fecundity in the population^10^. We assume that the stock-recruitment relationship is of the Ricker^11^ type. Such a type of stock-recruitment relationship is an appropriate description of recruitment biology of cod^12^. According to the Ricker model^11,13^, the development of the early-life history follows , where $N(0)=N_{0}$, and recruits enter the fish stock at $T=1,3$ years, respectively, depending on the fish stock. Natural mortality is made up of three components. Ocean acidification causes a higher larval mortality rate. This leads to a density-independent mortality rate *a/T* caused by acidification. Furthermore, *b*/T is the density-independent mortality rate at baseline conditions, and is the density-dependent which increases with the spawning stock, e.g. because of cannibalism (5). Solving the differential equation, we obtain

Where R denotes recruits in numbers, and . In the baseline-scenario, we have $a=0$, in the acidification scenarios, $e^{-a}$is the fraction of cod in the early life history stages that survives the effect of acidification. We use the data from experiments to quantify this effect.

To estimate the stock-recruitment relationship for the baseline scenario we use ICES data for Western Baltic cod for the years 1970 to 2014 and for the Barents Sea cod for the years 1946-2014. We assume log-normal auto-correlated errors, and estimate the model

,

where $\xi_{t+1}=\nu\xi_{t}+\varepsilon_{t}$, and $\varepsilon_{t}$ is a series of iid random variables. We obtain estimates= 0.929 with 95% confidence interval [1.05; 0.808] and -1.219/million tons with 95% confidence interval [-0.999; -1.439]/million tons for the Barents Sea cod as well as = 0.888 with 95% confidence interval [1.224; 0.553] and 4.762/million tons with 95% confidence interval [-4.672; 14.196]/million tons for Western Baltic cod.

## **SI References**

1. Riebesell U, Fabry VJ, Hansson L, Gattuso J-P (2010) *Guide to Best Practices in Ocean Acidification Research and Data Reporting* doi:10.2777/58454.

2. Dickson AG, Sabine CL, Christian JR (2007) Guide to best practices for ocean CO_2_ measurements. *PICES Spec Publ* 3.

3. Pierrot D, Lewis E, Wallace DWR (2006) MS Excel Program developed for CO_2_ system calculations, ORNL/CDIAC-105. Carbon Dioxide Information Analysis Center, Oak Ridge National Laboratory, U.S. Department of Energy, Oak Ridge, Tennessee, U.S.

4. Dickson AG (1990) Standard potential of the (AgCl(s) + 1/2H2(g) = Ag(s) + HCl(aq)) cell and the dissociation constant of bisulfate ion in synthetic sea water from 273.15 to 318.15 K. *J Chem Thermodyn* 22:113–127.

5. Mehrbach C, Culberson CH, Hawley JE, Pytkowicz RM (1973) Measurement of the apparent dissociation constants of carbonic acid in seawater at atmospheric pressure. *Limnol Oceanogr* 18(6):897–907.

6. Dickson AG, Millero FJ (1987) A comparison of the equilibrium constants for the dissociation of carbonic acid in seawater media. *Deep Sea Res Part A Oceanogr Res Pap* 34(10):1733–1743.

7. Le Quesne WJF, Pinnegar JK (2012) The potential impacts of ocean acidification: scaling from physiology to fisheries. *Fish Fish* 13(3):333–344.

8. ICES (2014) *Report of the Baltic Fisheries Assessment Working Group (WGBFAS), 3-10 April 2014* (ICES HQ, Copenhagen, Denmark).

9. ICES (2014) *Report of the Arctic Fisheries Working Group (AFWG), 2014, Lisbon, Portugal*.

10. Hilborn R, Walters CJ (1992) *Quantitative Fish Stock Assessment - Choice, Dynamics and Uncertainty* (Kluwer Academic Publishers).

11. Ricker WE (1954) Stock and Recruitment. *J Fish Res Board Canada* 11(5):559–623.

12. Cook RM, Sinclair A, Stefánsson G (1997) Potential collapse of North Sea cod stocks. *Nature* 385(6616):521–522.

13. Quinn TJ, Deriso RB (1999) *Quantitative Fish Dynamics* (Oxford University Press).
